# The contribution of early science education in developing children awareness of carbon footprints

**DOI:** 10.1038/s41598-025-34469-3

**Published:** 2026-01-07

**Authors:** Ali A. Al-Barakat, Rommel M. AlAli, Sarah B. Alotaibi, Tahani M. Alrosaa, Ali K. Abdullatif, Ashraf M. Zaher

**Affiliations:** 1 Department of Education, University of Sharjah, Sharjah, 27272 United Arab Emirates; 2 Faculty of Educational Sciences, Yarmouk University, Irbid, 21163 Jordan; 3 The National Research Center for Giftedness and Creativity, King Faisal University, Al-Ahsa, 31982 Saudi Arabia; 4 College of Education and Human Development, Princess Nourah Bint Abdulrahman University, Riyadh, Saudi Arabia; 5 Department of Arabic Language, College of Arts, King Faisal University, Al Ahsa, Saudi Arabia; 6 Translation, Authorship and Publication Center, King Faisal University, Al-Ahsa, 31982 Saudi Arabia

**Keywords:** Sustainability, Science education, Childhood, Carbon footprint, Environmental education, Education, Environmental social sciences, Environmental studies

## Abstract

This research explored how early childhood science education contributes to developing children’s understanding of sustainability, with a particular focus on the concept of the carbon footprint as a tangible indicator of environmental impact. Using a qualitative design, semi-structured interviews were conducted with 33 award-winning science teachers from Saudi Arabia, the United Arab Emirates, and Jordan, while data was analyzed through grounded theory methodology. Findings revealed that effective integration of sustainability in early science education is achieved when teachers contextualize the carbon footprint concept through everyday classroom activities, link science lessons to real environmental issues, and engage children in experiential learning such as energy-saving practices and waste reduction. The research also revealed that sociocultural factors influence teachers’ implementation of sustainability concepts, highlighting both innovative practices and structural challenges. The research contributes to providing an empirically grounded framework for embedding sustainability and carbon footprint education in early childhood curricula. It recommends designing teacher training programs that equip teachers with practical, developmentally appropriate strategies to promote environmental culture and sustainable behavior from an early age.

## Introduction

One of the recent environmental challenges is climate change, since human activities build up greenhouse gases which raise the temperature, frequency of extreme weather events, reduce biodiversity, and, therefore, threaten the sustainability of life on Earth^1,2^.

Carbon footprint is a tool that quantifies the amount of carbon emissions that an individual or community generates, it is at the core of how humans’ daily habits contribute to worsening climate change. In terms of Early Childhood Education (ECE) perspective, introducing children to the idea of the carbon footprint is a considered way to educate them on the short-term impact of their simple actions on the environment^3,4^. Such simple actions, including walking instead of driving, switching off unnecessary lights, saving energy and water, or recycling assist them to make a direct link between their own behaviors and their carbon emissions. Hence, early childhood education is a powerful tool for promoting children’s environmental awareness and sustainability through actual events and providing opportunities to comprehend the extent to which their daily actions affect climate change^5,6^.

The Sustainable Development Goals, including Goal 13 on climate action, are specific efforts to reduce carbon and develop climate-resilient societies. So, early childhood Education is an important element of the sustainable decision-making process that children need to differentiate between environmentally friendly daily behaviors and those that are potential to have a greater environmental footprint^2,5^. Preschool and primary school children are able to easily conceptualize basic topics of the environmental impact and carbon emissions (carbon footprint, water usage, waste sorting, recycling) when these topics are delivered in a real-world context and are embedded into the action of taking control of real items, such as measuring electricity use in household, tracking water consumption, and sorting refuse^7^. Experiencing these issues directly gives children an awareness of the links between their actions and the consequences on their environment and, accordingly, take responsibility for making more mindful choices about their environment.

School settings can offer critical experiences for the environmental identity that represents the sense of understanding and belonging to nature for young children^8,9^, including a sense of environmental responsibility for natural resources and environmental behavior^10–13^. Such settings give children the chance to have direct exposure to the environment and understand how they can positively contribute to the natural order they live in through a multi-sensory learning approach, such as observing the growth of plants, observing animals in the surrounding environment, participating in recycling initiatives, and calculating energy and water consumption^14–16^.

The teacher is at the forefront of the young age-environmental learning process which encompasses children’s understanding of carbon footprint, his role directs hands-on experiments and direct observation of the repercussions of environmental behaviors^17–20^. Common teachers’ methods of active learning include experimentation on, for example, energy, water, and waste as a way to educate children on how their everyday choices affect the environment, for instance, an experiment could compare the consumption of electricity of two different household appliances or the growth of one plant when water-saving irrigation methods are applied. Such activities develop scientific inquiry and critical thinking, help children grasp cause-effect relations in a tangible way, allow linking learning to their real environments, and support them to promote sustainable practices like: resource conservational, waste minimization, and sustainability-oriented transportation^21,22^.

The carbon footprint is an efficient means of making human impact on the environment an elementary classroom topic for children^23–25^. It is defined as the total greenhouse gas emissions generated by an individual or group behaviors, such as transportation, energy usage, and food preparation^9,26^. The demand for plant and food products and fibers, livestock and fish products, and wood products, as well as the amount of land for urban infrastructure and for forests required to absorb carbon dioxide emissions generated by fossil fuels measures the carbon footprint of an individual or community^27^.

Stories, pictures and interactive videos on daily activities are also associated with higher levels of emissions, they can help to simplify this concept for children. As a result of this hands-on approach, children are able to view the impact of their actions in real life and how they can act to reduce their impact over time. Examples of comparative experiments that highlight the discrepancies of low- and high-emission activities can develop early understanding of the individual behavior towards the environmental impact 28,17].

A personal footprint is the direct impact on the environment of how and where people use resources such as foods, transportation, goods and services, it is determined by how people live, what they like to have, and how much they use resources^28–30^. With growing awareness of environmental concerns, education of children about the effect of carbon emissions caused by daily activities is more crucial than ever. The carbon footprint is the quantity of carbon dioxide (CO₂) gas emitted through energy consumption, transportation, food production, and other human activities, making it an important indicator of an individual’s and society’s environmental impact. World Wildlife Fund (WWF) stated that countries can quantify and monitor their environmental assets using ecological footprint calculations, and it is also vital to engage citizens from an early age in promoting their awareness and participation in the reduction of resource consumption and carbon emissions^29^.

The carbon footprint includes every natural element that serves the activities of individuals and communities, including energy, food, rangelands, forests, marine areas, and built-up areas, as they are needed to absorption of carbon dioxide resulted from energy use, food production, animal products, timbers, fish; and the land needed for residential areas and infrastructures^3,4^.

Recent researches showed that carbon footprint measurement tools can affect environmental awareness in young age and help to create a more sustainable generation^27^. Baldwin et al.^28^ reported that the utilization of such tools in the learning process provides teachers an effective pedagogical approach which integrates information, participation and empowerment, and therefore improves children’s environmental awareness and understanding of their responsibility for conservation. Moreover, Alali et al.^31^ reported that carbon footprint is an educational resource to teach children about sustainability, since it makes their esoteric environmental values come up into actual activities and real-world ties, and so, a generation is raised ready for making mindful, meaningful, sustainable environmental choices.

Numerous researchers^11–13,32–35^ argued that practical experience and daily observation are of the most effective methods to teach about carbon footprint in children, while Wiedmann & Minx^36^ suggested integrating carbon footprint within a framework for environmental knowledge education through daily behaviors in a sustainable and realistic way, as children discover, by doing things as simple as calculating what effect each travel, food or energy use has on the environment, that every day decisions make a difference. The teacher leads to environmentally friendly decisions through group projects that encourage them to think critically and engage in conscious attitudes towards the environment, which, in turn, develop sustainable positive environmental practices that last for lifetime^37^.

Research on education^31,38–40^ demonstrated that youth who were introduced to carbon footprint ideas at early age undergo more substantial change in environmental behavior and develop a sense of responsibility to nature. They are engaged in the consumption of electricity and water monitoring, environmentally friendly transportation, awareness of the consequences of personal decisions, and motivation to engage in environmental projects such as public outdoor littering and tree-growing programs^41,42^. They learned to deal with environmental issues and think and to act on fewer negative environmental outcomes, thus keeping sustainability in their lives^20,27,43^.

Embedding carbon footprints into early childhood curriculums enables students to work through abstract concepts in a practical way and develop skills in sustainable behavior^44–51^. Discussion within the classroom on carbon footprint science activities has an impact on building collaboration, creating a sense of social responsibilities, engagement in community, and realizing the position of the child to protect the environment through environmental initiatives^9,52,53^. Teachers can help the children keep track of their home energy and water usage, calculate the daily amount that goes into the garbage, and share the effect of different methods of transportation with them. Recycling initiatives and planting mini-gardens at school and other group activities may, also, be included, enabling environmental ideas to be better taught and linked to everyday life^44^.

Accordingly, early childhood education is regarded as a strategic mechanism that encourages sustainable environmental processes, critical thinking, and responsible decision making^38,54^. Currently, activities that incorporate carbon footprint and sustainability are not systematically conducted, and do not connect concepts to real-life situation^11,35,43,55,56^. So, practical learning must be integrated into curricula through measuring resource use, sorting waste, and carrying out collective environmental projects that turn studying into a hand on, real-life experience, since such methodology encourages kids to build critical thinking, environmental decision-making behaviors, and builds up the sense of being responsible for environmental impact as individuals and communities, so they know how they can start to take an active role in building a sustainable world from a young age.

Earlier research has emphasized early childhood education as key factor to establish environmental protection, many of them focused purely on theoretical frameworks or classroom-based activities, with little discussion of teachers’ views on how these concepts can be embedded in classroom practice. The novelty of the research and its originality can be summed up in the following aspects:

1. Focusing on the Teachers’ Perspective: While earlier studies focused on children’s learning, this research investigates teachers’ perceptions of the necessity of environmental education and the application, thereof, to the early grades. It enables a more effective grasp of the critical barriers to the systematic application of environmental and carbon footprint concepts into the educational context, along with identifying key hurdles to sustainability and climate education.
2. Linking between the Importance of Teachers’ Perceptions and Practice: By contrasting teachers’ actual practice, as described, to their perception of value for environmental subjects, the research discloses the inconsistencies between teachers’ environmental beliefs and goals and how these are actually carried out within the classroom. It also guides teachers to identify opportunities for continuing development (or updating the curriculum) in proportion with current ecological standards and trends.
3. Assessing Practical Applicability: The research assess teachers’ perspectives on incorporating environmental topics into realistic classroom settings, since these perspectives are necessary for effective implementation of sustainability and carbon footprint concepts within learning environments, as well as exploring potential obstacles such as time constraints, resource limitations, and lack of institutional support.
4. Enriching Curriculum Design: Based on teachers’ practical experiences, the research provides practical evidence that guide the design or modification of early childhood education programs towards enhancing children’s environmental awareness to bridge the gap between theory and practice, making environmental education more effective and feasible and promoting sustainability of teaching environmental concepts.
5. Investigating the Carbon Footprint: This research is one of the few studies that have addressed behaviors related to the carbon footprint in early childhood education. Moreover, it may be among the first studies to examine this issue in Arab educational settings.

Despite the emphasis on the importance of integrating sustainability principles into education, many early childhood curricula in Arab countries still lack the effective integration of environmental concepts within science education^38^. Accordingly, teachers remain the key link between educational theory, policy and practice through transforming theory into real-world experiences that fit children’ unique developmental characteristics through sensory events, communal projects, and observational and discovery-based learning.

This research aimed at exploring the perception of outstanding teachers towards integrating carbon footprint topics into early childhood science education as well as analyzing their understanding of its educational value and its role in building environmental awareness among children. Besides, the study assumes that teachers’ perceptions reflect the educational and cultural environment and national policies that frame early childhood education practices rather than being merely personal opinions. The research, based on this perspective, contributes to bridging a knowledge gap in the literature on environmental education, since most earlier studies neglected the perspectives of teachers and focused on children’s learning outcomes or curriculum analysis. Also, the research sought to highlight the teacher’s role as a facilitator of environmental awareness and a guide for children’s behavior towards more sustainable practices, based on the premise that building “sustainable citizens” begins from the very first moments of learning. Accordingly, the study is based on the following main research question: “H*ow do experienced teachers perceive importance*,* practice*,* and feasibility of integrating environment-related topics in early childhood education?”*

## Methodology

### Research design

A Grounded Theory-base qualitative approach was adopted to explore how early childhood science teachers develop environmental awareness among children, particularly in terms of carbon footprint, since Grounded Theory aims at developing a theoretical framework that explains processes, actions, or interactions based on actual data. This approach allowed authors to systematically analyze teachers’ perceptions interactions, and strategies to build a conceptual model that illustrates how sustainability concepts are integrated and applied within early childhood classrooms, and, thus, provided a deeper understanding of how early science education influences the development of environmentally responsible behaviors in children.

### Participants

The research sample included 33 science teachers who were working in kindergartens and primary schools in Saudi Arabia, Jordan, and the United Arab Emirates, since educational policies in these countries include clear directives to teach children environmental protection and sustainability concepts from the earliest stages of schooling (the Saudi primary science curriculum includes activities on energy, water, and recycling, the Jordanian curriculum integrates climate change concepts, and the Emirati curriculum encourages practical environmental projects). Voluntary purposive sampling technique was used, all participants have submitted official documentation demonstrating that they had won Educational Excellence Awards granted by ministries of education or official educational institutions according to specific criteria, including the quality of teaching performance, the integration of environmental concepts into the curriculum, and the development of engaging educational activities for children in the field of sustainability.

Twelve teachers were selected from Saudi Arabia (36.37% of the sample), ten from Jordan (30.30%), and eleven from the UAE (33.33%). These teachers were identified based on their academic and pedagogical excellence and their accreditation by school administrations and educational institutions as teachers who incorporate sustainability and carbon footprint concepts into curricula, as well as their ability to motivate children to think about environmental protection.

The sample was deemed sufficient based on reaching the saturation level; this was achieved once it had been established from the available data that it had reached diversity in terms of experiences and opinions related to teaching sustainability and carbon footprint by teachers. Continuous monitoring of all data from responses related to teacher experiences aimed to determine if saturation had been reached; this would be apparent if it had been established that no new information had been gathered from additional data collection exercises and if there were redundancies in responses based on perceived experiences. The redundancy of data in qualitative research would indicate completion of data collection efforts and would indicate satisfaction with data diversity related to teacher experiences to ensure research reliability.

### Data collection tool

Semi-structured interviews were used for collecting qualitative data due to their ability to focus on teachers’ actual classroom experiences. This helped in understanding how teaching sustainability concepts including carbon footprint impacts children’s environmental awareness and behaviors. The authors conducted these interviews after receiving standardized training in interviewing techniques. The authors followed a standardized question series to ensure common methodology in answering questions by all teachers. The recordings were periodically assessed to ensure consistency in following- up responses to questions by all researchers and to improve the validity of results. The interviews had four open-ended comprehensive questions related to the theory of teaching carbon footprint in early science education. The drafted follow-up questions to clarify responses if there were misunderstandings about teaching carbon footprint in science education.

The initial version of the interviews was reviewed by a committee of seven experts in early childhood education, environmental education, and qualitative research analysis, where their feedback led to simplifying the language of the questions and restructuring them to move from general concepts to practical classroom application. The reviewers also developed flexible follow-up questions that enabled authors to collect accurate and comprehensive data.

Moreover, the instrument was piloted with five qualified teachers outside the main sample to test the clarity and ease of the questions and to assess their ability to elicit accurate information about actual teaching practices. Accordingly, there was a need for some modifications, including improving the transitions between questions, rewording the first question to include situations where the concept of carbon footprint is not directly introduced, and removing or modifying questions that relied on the teacher’s personal opinion, this, in turn, helped collect richer and more diverse data. The final version of the interview schedule is presented in Table 1.

Table 1 presents the semi-structured interview questions, which include four main questions and eight follow-up questions. This means that each main-question accompanied by two follow-up questions.

**Table 1:** Interview guide questions.

No.	Main question	Follow-up questions
1	How do you introduce the concept of carbon footprint to children in your class?	If this concept is not presented, what other environmental or behavioral concepts do you use? And how do these concepts relate to reducing the environmental impact of children?
2	What activities or examples do you use to help children understand the concept of carbon footprint??	Can specific examples be provided from the classroom or from daily activities in which the children participate? How do you observe the children’s interaction with these activities?
3	What daily behaviors can children be encouraged to practice reducing environmental impact?	How do you explain the relationship between these behaviors and the concept of carbon footprint? What changes have been seen in the children’s behaviors because of these practices?
4	How do you observe changes in children’s awareness of environmental concepts and carbon footprint throughout the academic year?	Are there any tangible indicators of changes in children’s behavior? How are these changes monitored and followed up within classroom activities?

### Data analysis

In order to effectively grasp the real-life teaching practices and experiences of teachers and relate them to general educational objectives of instilling environmental responsibility, data collection was systematically conducted to provide insight into how early science education impacts environmental knowledge, carbon footprint awareness, and sustainability behavior in children. Firstly, two separate researchers thoroughly evaluated all the transcripts to have a general idea and understanding of teacher responses. The evaluation helped to note critical statements and pointers related to research objectives.

The data analysis process went through a multi-stage coding procedure starting with an open coding stage where each paragraph of the coded transcripts was labeled with descriptive code that captured key ideas, classroom practices, and teachers’ observations related to environmental education and carbon footprint, this helped extract rich and detailed data without imposing predefined categories, ensuring that participants’ perspectives were accurately represented.

Next, pivot coding was applied to group related codes into broader conceptual categories that reflect patterns and relationships. The categories were aligned with the research objectives to provide a framework that help understand how teachers integrate carbon footprint concepts into early science education.

**Fig. 1:**
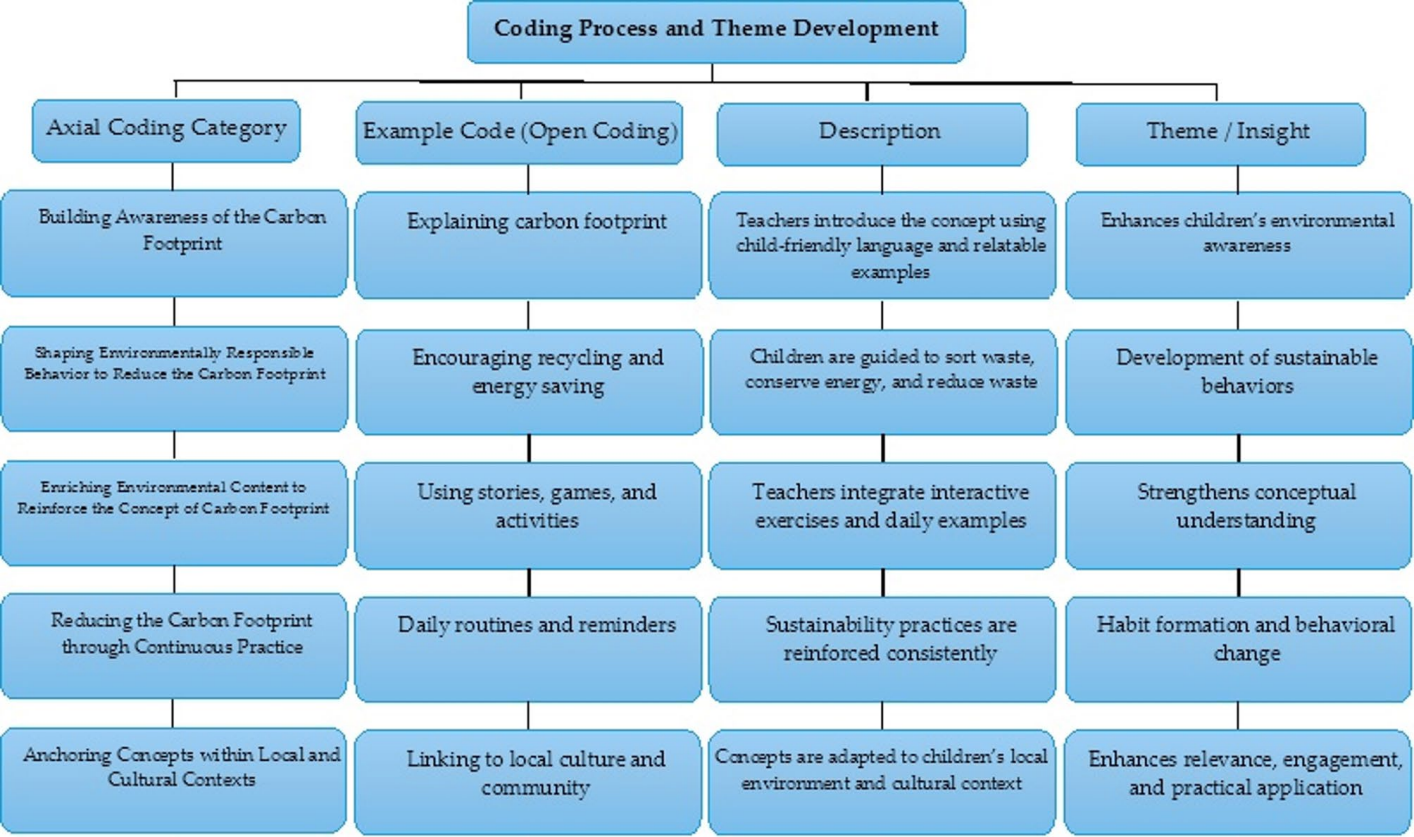
Illustrates the method and the stages through which the interview data analysis process was conducted.

Figure 1 illustrates the relationship between open codes, pivot categories, and insights, through presenting selected examples of how raw data was systematically analyzed to develop key themes, which, in turn, demonstrates the transparency and accuracy of the coding process and shows how teachers’ responses contributed to the study’s conclusions.

### Ethical consideration

Ethical issues and participant rights where considered across all research phases, as authors obtained ethical approval from the Research Ethics Committee at King Faisal University, which in turn contacted the Arab Bureau of Education to facilitate the participants’ engagement due to the Bureau’s direct relationship with the participating teachers. Besides, all participants were fully informed of the research objectives and procedures, provided with detailed information about their rights including the right to withdraw at any time, and the informed consent was documented to ensure respect for participants’ rights and dignity. Participant confidentiality was ensured through the use of pseudonyms during analysis and reporting, the removal of any identifying information such as names and schools, and the secured storage of data with strict access controls. This reflected the commitment to ethical principles of honesty, transparency, and respect for participants’ rights, fostered trust and comfort among participants, and encouraged them to provide truthful data and genuine participation.

## Results of the research

Data analysis showed the following five significant major categories: building awareness of the carbon footprint, shaping environmentally responsible behavior to reduce the carbon footprint, enriching environmental content to reinforce the concept of carbon footprint, reducing the carbon footprint through continuous practice, and anchoring concepts within local and cultural contexts. The categories are summarized as follows:

### Building awareness of the carbon footprint

The data analysis demonstrated that teachers are the primary change agents who guide children in developing the understanding of the environmental concept of carbon emissions, as 31 participants (93.9%) emphasized that awareness of carbon footprint during early childhood teaching is a primary building block to building early awareness of the environment which is necessary for adopting sustainable behaviors that support the reduction of emissions. Many participants expressed that this type of awareness could be raised with the conceptualization and implementation of actions that bridge the gap between science learning and children’s everyday life situations. As most participants indicated, science education in the early years is critical for nurturing the environmental awareness and stewardship, whereby children learn, for instance, that their actions such as using electricity or littering have negative impacts on the environment. A teacher stated:

There seems to be a void in the professional training on promoting children’s environmental footprint, teachers think of it as simply teaching science, but it is nurturing an ecological mindset. When I explain that plants can purify air, I notice some children control themselves from picking flowers or breaking branches, this is where science becomes a sentiment.

The educational approach to teaching science content to children enables their emotional development because it enables them to understand their environmental role through developing emotional bonds with nature which transforms environmental elements into personal values instead of academic requirements. A science teacher explained that teaching science to children requires preserving environmental values, stating:

The core purpose of teaching science to children should involve teaching them values, as this learning will affect both their existence and environmental relationships. During teaching, I show students how greenhouse gas emissions relate to home electricity usage and car driving.

The integration of scientific learning into daily routines allows children to deepen their understanding of how personal decisions affect major environmental issues such as climate change and global warming. A child understands how running an unattended light produces carbon emissions, even though its amount does not matter, this recognition leads to self-regulation which enables him to move past awareness toward practical sustainability.

### Shaping environmentally responsible behavior to reduce the carbon footprint

The data analysis demonstrated that children aged 3 to 6 years are at the most optimal age for learning how to reduce carbon footprint, since 30 participants (90.3%) considered children of this category as the best respondents for educational practices that promote environmental values and behavior. Those children develop foundational learning, eco-literacy, and environmental awareness as they learn to imitate and engage directly with their environment. The established daily routines transform scientific knowledge into actual practices. A teacher explained this by stating:

Children at this age stage merge their classroom learning with their everyday experiences, they demonstrate immediate action after classroom discussions about water conservation as they practice turning off the tap during toothbrushing in the following day. This immediate response becomes less noticeable when children grow older.

The above statement supports the idea that education should not be treated as mere information given and received in a classroom, instead, it should be considered as a dynamic, active practice that shapes learning. Science education transforms children into hands-on practitioners who create sustainable habits through practices. A teacher explained how practical assignments help develop behaviors among both children and their surrounding community, stating:

Grade four students established the “Turn off the Lights” campaign after finishing their lessons about various energy sources. They knew that electricity usage involves expenses and climate effects due to classroom discussion. Education reaches its highest level at this point because it transforms theoretical knowledge into actual practice.

The development of scientific knowledge among children shows promising results because it creates shared responsibilities and environmental activism within families and educational institutions. The learning process requires more than memorizing facts because students must comprehend resource usage effects on individual and group carbon emissions.

### Enriching environmental content to reinforce the concept of carbon footprint

The analysis of data demonstrated that 30 participants (90.9%) confirmed that children receive lessons about carbon footprint as well as activities related to fundamental environmental subjects including renewable energy, recycling, natural resource protection, and climate change awareness. The inclusion of these environmental concepts in school curricula creates a positive base for teaching children about their environmental impact through daily activities and carbon footprint knowledge. Despite that, the participants reported that the presence of rich environmental content supported by science concepts does not achieve the intended change in awareness. The results mainly rely the way of presenting the concepts and the extent of the teacher’s involvement in learning activities. A teacher, talking about her experience while teaching “Natural Resources” unit, said:

When I start teaching about renewable and non-renewable energy, I get the question ‘Why don’t we always use solar energy?’ This conversation is where their mindset changes a lot regarding electricity use.

Seeing children’s ability to think critically about their behaviors that mitigate their carbon footprint is a testament to the influence of this curriculum content. Shifting from receiving information passively to being active participants in evaluating the environmentally friendly choices is the beginning of children analytical and critical eco- awareness. The depth of the impact of the content is further enhanced with active practice where children use their knowledge thereby creating a strong bond between theory and practice. One teacher emphasized the Value of the Recycling lesson as follows:

Teaching the Recycling lesson at school is easy, we organize an in-class exhibition where students bring recyclable materials, after this activity, I notice that the children stopped throwing paper in regular waste bins and even started doing it at home.

Here, the focus is on active participation that is necessary for learning controlling wasteful behavior which is most effective in reducing the carbon footprint. The ability to demonstrate the role of waste to reinforce sustainable education is most powerful when the behaviors are practiced outside the classroom. In this respect, the use of educational videos with interactive story elements activates children’s emotional and intellectual resources to promote environmental care.

### Reducing the carbon footprint through continuous practice

The findings demonstrated that the repetition of environmental initiatives and activities that directly reduce carbon footprint such as trimming down energy and water usage, waste reduction, and recycling is crucial to developing sustainable environmental behavior among children. Twenty-nine participants (87.8%) reported that reinforcing the sequence of activities innovatively enables the children to progress from fleeting behavioral responses to daily firmly established environmental habits. One teacher expressed this saying:

We innovatively repeat the recycling project every year. Firstly, it was an educational activity, but now it has become a part of the classroom culture, older children promote recycling and encourage newer ones not to throw away paper.

The quote illustrates that, in this context, repetition is constructive as it enriches the experience of children and reinforces their connectedness to a school community with environmental values. Continuous repetition of environmental activities gradually shifts the child from being a passive recipient to an environmentally proactive person. It also reduces waste and the carbon footprint. Regarding this issue, one participant stated:

Every week, we hold an ‘Environmental Day’ where children select a topic and carry out a simple, associated activity. The repetition profounds awareness as alternative, environmentally friendly options become the obvious and habitual choice.

This quote describes the ‘spontaneous environmental awareness’ of children, where considering the environmental impacts and the associated carbon footprint of daily activities become automatic, and their positive environmental behavior requires no external prompting. The most profound form of awareness is also the most sustainable, because it is a result of a dispersed, repeated experience and underlying convictions. Another participant said:

Every end of every term, I assign my students the task of writing down what was the most committed environmental behavior they adhered to, they sometimes recall that they turned off the lights or picked up litter during an outing. These reflective exercises demonstrate the impact of exposure to experience on the children’s behaviors.

The activities prompted the children to understand the impact their actions on the environment and the resultant carbon footprint of their activities. Deep environmental education is built on teaching responsible self-awareness which can be achieved through activities and experiences rather than theories.

Integrated environmental education activities sustain both knowledge and cultivating behavioral habits aimed at carbon footprint reduction. Based on theories of recycling and energy economy, the child practice sustainable and positive differentiation in both home and community environments, possesses and understands the concepts of environmental protective engagement, and succeeds in transferring knowledge to peers.

Continuous environmental activities and self-initiated learning shape the child’s “early environmental identity” which embeds a psychological connection with the environment to adopt practices that reduce carbon footprint. Early scientific education contributes to knowledge transfer as well as values and behaviors, creating a generation that understands the climate crisis at personal responsibility level.

### Anchoring concepts within local and cultural contexts

Children’s environmental understanding and mastery of sustainability actions can be better developed when carbon footprint and environmental concepts are taught within local and culturally relevant contexts. Twenty-seven participants (81.8%) reported that when students learn environmental concepts through real-life issues, such as resource consumption at home, transport in their neighborhood, and family spending habits, students are more responsive and able to process the information. Connecting scientific knowledge to personal experience not only enhances understanding but also cultivates a sense of ownership and belonging to the environment. This was best expressed by a participant who said:

When I addressed the topic of plastic waste, I asked the students to record the number of plastic bags they use weekly at home. They were astonished by the quantity of the consumption and began discussing more sustainable alternatives with their families because the lesson resonated with their daily lives and became personal.

This is a good example of how scientific knowledge can give a child the mental framework that strengthens personal beliefs and the moral power to take action to reduce carbon footprint through household practices. Children learn to shift from receiving information passively to actively change their surroundings. This was illustrated further by another participant who expressed how studying the local culture improves the educational environment:

A child asked about riding a bicycle in our neighborhood during an educational video, we started talking about safety customs, traditional weather patterns, and protocols in our country, the lesson then shifted to a dialogue about similarities between community and environment.

An educational environment provides children with the opportunity to actively consider and critique the social and environmental standards and constructs of their local surroundings. Student dialogues cultivate “environmental citizenship” as they channel critical thinking in situational behaviors of their community. Another teacher’s experience exemplifies how powerful the effect is when children express something personally.

After asking students to describe how their families can reduce their carbon footprints, I received some wonderful stories that included eating habits and types of transport. The children began to recognize that both the problem and the solution rested with them and their families.

This method appeals to the emotional and cognitive processes of the children, hereby, they become “environmental narrators,” converging their experiences and knowledge to deepen their commitments. The role of families in learning ensures that activities in school reach other parts of the community. The cultural elements and norms play major roles in shaping the concept of environment among children. The adoption of education in their activities makes it relevant and results-oriented; this helps the children grasp the impacts of their actions in their environment and inspires them to act towards responding to issues pertaining to their environment.

Schools provide an educational environment where students acquire skills to make environmentally responsible decisions depending on their context and, in turn, encourage positive environmental awareness and eventually a marked reduction in carbon footprint, which is a fundamental requirement met by childhood education.

## Results discussion

The results revealed that science learning in early education plays a critical part in awareness of the environment and sustainable behavior as well because kids discussing “carbon footprint” and effects of their activities on the environment helped to form a basic understanding of what they do to their environment. The role of teachers in such direct learning from their classroom activities related to these learning concepts should be considered because kids’ knowledge from science was related to their behavior. For example, their science learning related to their usual life activities helped kids to comprehend what they do by very small things such as switching off the lights and disposing of paper properly.

The findings also unveiled some remarkable environmental practices of children, like switching off lights, water saving, etc., after a few lessons which indicate that the learning process of the kids was not only academic but also practical. This highlights the role of teachers continuing working with organized activities that not only engaged children cognitively, emotionally, and socially but also made them feel committed to the environment and responsible for it. These results are in line with the previous studies^10,21,57,58^, which pointed out that children not only gain knowledge but also values mainly through the observing and imitating of the models showing ethical and responsible behavior.

The results also showed that when children’s environmentally responsible behaviors were reinforced, teachers continued to carry out environmental activities in the classroom. This is likely because the curriculum was designed in a way that matches children’s developmental needs. As children repeatedly engaged in these activities every day, their awareness gradually turned into actual habits. Moreover, the support children received in both the classroom and the community helped these positive behaviors extend beyond school and influence practices at home, highlighting the wider social impact of early science education.

Sihvonen et al.^52^ showed that hands-on activities in early childhood - such as managing energy and water use and reducing waste - can help lower children’s environmental footprint. This aligns with what was observed in the present study. While these findings support previous research, this study adds a new dimension by highlighting the role teachers play in embedding environmental education within the local cultural context.

In addition to this insight, it was clear from these results that knowledge about the environment becomes even relevant if it leads to engagement and relates to what happens in children’s day-to-day life. The reason early science learning interventions have been shown to have value in promoting sustainability behavior can be attributed to their ability to connect knowledge to emotional engagement and practical application. Children not only absorbed new knowledge but made their own connections to nature, which helped build their emotional ethics of responsibility to make rational decisions. For example, it was seen from these results that after teaching them about saving water in their environment, they were all reminding each other not to let water run from their taps and even started suggesting using their leftover drinking water to water their planted garden.

The results have also shown how early environmental interventions have effects not only in the classroom but also in the family and community context. This can be facilitated by the presence of the teacher as an intermediary who uses methods of teaching in line with the culture and traditions of the community. The example here would be to ask these children to monitor plastic usage in their families and to discuss ways to make their transport ‘eco-friendly.’ These kids would be agents of change as they would educate others in their families about what they have learned.

The result supported the finding of previous studies^34,35,45,59–61^, which stated that hands-on actions in kindergartens related to energy and water management, reduction of waste, as well as encouragement of active transport contributes to ‘the effect of children’s behavior towards their families. The result can be justified due to the value of ‘experiential learning and hands-on involvement in understanding sustainable actions by children to analyze their families’ behavior and come up with solutions.’ The result uncovered a strong linkage between knowledge and actions of sustainability by combining knowledge and involvement in children’s lifestyle. The result supports the finding of research conducted by Al-Barakat et al.^38^, which validated that interactive learning and hands-on involvement result in actual changes to their behavior towards the environment.

In conclusion, the results represent the effect of children on their families and communities; because of the large number of children who encouraged their families to cut down their use of plastic materials, it represents the effectiveness of environmental education conducted among members of the community. The reason for this effectiveness can be attributed to several interactive elements such as gaining teacher cooperation and support, offering culturally relevant educational materials related to sustainability and environmental issues related to their culture and community, numerous hands-on activities conducted among these communities, and their emotional connections to make them go from being passive learners to being active members who have the ability to make sound and responsible decisions to decrease their carbon footprint either individually and collectively.

The findings provide valuable insights into the impact of early science education on developing environmental awareness and promoting sustainable behaviors among children through highlighting the role of integrating concepts such as “carbon footprint” into everyday classroom activities in enhancing children’s understanding of the environmental consequences of their actions. However, critical analysis also reveals variations in the extent to which teachers successfully integrate knowledge and behavior, as some were able to incorporate carbon footprint concepts into ongoing classroom practices while others faced difficulties stemming from a lack of specialized training or weak institutional support. These findings align with^21,37^ who identified organizational and cultural challenges as major obstacles to implementing sustainable environmental education.

In general, based on these results, it is clear and apparent that in order to receive quality environmental education in early childhood education, it would be necessary to have comprehensive learning approaches encompassing theoretical knowledge and application skills, and added to these requirements would be needed a conducive environment for learning to occur from the teachers’ perspective because it not only deals with imparting knowledge about environment to these persons but would also require them to act as catalysts to build critical knowledge out of their self-learning experiences to make these kids into responsible human beings who make responsible choices not only individually but also together as society as whole. Added to these requirements, these results have confirmed to have dual learning effect to accomplish related to application of carbon footprint concept in early science education.

The results of this study highlight the need to conduct additional comparative research to assess the effect of cultural and structural variations across different regions internationally regarding the application of environmental education and ultimately to construct a comprehensive global framework regarding teaching EC to preschoolers.

## Conclusions, recommendations, limitations, and future research directions

The study helped to provide insight into early childhood teachers’ approaches to incorporating environmental sustainability and carbon footprint awareness into science education by identifying their critical role as productive facilitators who translate knowledge about the environment into real-life experiences for children to learn about science-related concepts in their own context. The study used semi-structured interviews involving 33 award-winning teachers from three different countries.

The study provides very relevant information about teaching methods and learning strategies for sustainability issues in early childhood education. The results show that it’s best to learn about the environment if teachers use experiential learning techniques such as learning about nature and community environment initiatives; such approaches to learning stimulate critical thinking and curiosity among kids.

The study also brought out the need for professional development for teachers to ensure they learn strategies to help imbue sustainable thinking skills and environment-related practical skills in children. The study also emphasized the importance of cooperation between school and society to ensure learning extends beyond the classroom environment and makes learning about the environment an integral part of society’s culture.

The authors state several limitations from their study that must be kept in mind if one were trying to draw conclusions from their findings. These would be data gathered solely from teacher responses to questions; such data may result in bias from trying to portray what would be considered an ideal scenario. Further bias arises from this study focusing solely on award-winning teachers who would portray their actions in what would be perceived as ideal scenarios not necessarily common to all teaching professionals. In addition to these issues, developing their study from only one research instrument would not allow cross-reference to be accomplished.

Considering the research findings, it is recommended to promote professional development programs that equip teachers with the skills to apply sustainability and carbon footprint concepts in interactive ways appropriate to children’s developmental age, such as experience-based activities, observation, and discovery. Also, it is recommended that curriculum developers should explicitly integrate sustainability concepts into science curricula and design learning units that encourage critical thinking and daily environmental-friendly practices, making environmental issues part of the continuous learning process. Furthermore, it is recommended that educational policymakers should integrate environmental education principles into national educational plans, provide institutional and financial support for implementing sustainability initiatives at schools, and encourage partnerships between ministries of education and ministries of environment to implement joint national programs that promote environmental awareness among children from a young age.

It is also recommended that future research be conducted with more diverse samples in terms of experiences, cultures, and types of educational institutions, and that quantitative or mixed methodologies be used to measure the long-term impact of environmental education. Besides, it is also recommended to employ educational interventions or pilot curriculum models through which assessing the effectiveness of environmental activities in promoting children’s awareness and sustainable behaviors is possible. Finally, it is recommended to explore the potential of employing modern educational technologies, such as interactive applications, augmented reality tools, and environmental educational games, to make learning sustainability concepts more engaging and effective.

## Data Availability

The authors will make the raw data supporting the conclusions of this article available upon request, without any undue restrictions. Requests for access to the raw data should be directed to Ali Ahmad Al-Barakat (aalbarakat@sharjah.ac.ae).
